# Influences of Stocking Density on Antioxidant Status, Nutrients Composition, and Lipid Metabolism in the Muscles of *Cyprinus carpio* under Rice–Fish Co-Culture

**DOI:** 10.3390/antiox13070849

**Published:** 2024-07-15

**Authors:** Yongrong Rong, Bing Li, Yiran Hou, Liqiang Zhang, Rui Jia, Jian Zhu

**Affiliations:** 1 Wuxi Fisheries College, Nanjing Agricultural University, Wuxi 214081, China; 2022813035@stu.njau.edu.cn (Y.R.); lib@ffrc.cn (B.L.); houyr@ffrc.cn (Y.H.); zhangliqiang@ffrc.cn (L.Z.); 2 Key Laboratory of Integrated Rice-Fish Farming Ecology, Ministry of Agriculture and Rural Affairs, Freshwater Fisheries Research Center, Chinese Academy of Fishery Sciences, Wuxi 214081, China

**Keywords:** stocking density, multi-omics, nutrient profile, metabolic functions, *Cyprinus carpio*

## Abstract

*Cyprinus carpio* is a significant freshwater species with substantial nutritional and economic value. Rice–carp co-culture represents one of its principal cultivation methods. However, in the system, the optimal farming density for carp and the impact of high stocking density on their muscle nutritional composition have yet to be explored. Thus, the objective of the current study was to investigate the influences of stocking density on the muscle nutrient profiles and metabolism of *C. carpio* in rice–fish co-culture systems. Common carp were cultured at three stocking densities, low density (LD), medium density (MD), and high density (HD), over a period of 60 days. Following this, comprehensive analyses incorporating physiological, biochemical, and multi-omics sequencing were conducted on the muscle tissue of *C. carpio*. The results demonstrated that HD treatment led to a reduction in the antioxidant capacity of *C. carpio*, while resulting in elevated levels of various fatty acids in muscle tissue, including saturated fatty acids (SFAs), omega-3 polyunsaturated fatty acids (n-3 PUFAs), and omega-6 polyunsaturated fatty acids (n-6 PUFAs). The metabolome analysis showed that HD treatment caused a marked reduction in 43 metabolites and a significant elevation in 30 metabolites, primarily linked to lipid and amino acid metabolism. Additionally, transcriptomic analysis revealed that the abnormalities in lipid metabolism induced by high-stocking-density treatment may be associated with significant alterations in the PPAR signaling pathway and adipokine signaling pathway. Overall, our findings indicate that in rice–fish co-culture systems, high stocking density disrupted the balance of antioxidant status and lipid metabolism in the muscles of *C. carpio.*

## 1. Introduction

As the global population expands and agricultural resources become increasingly scarce, humanity faces significant food security and malnutrition challenges [1,2]. In response, agricultural production has intensified over recent decades, with the excessive application of pesticides and mineral fertilizers exerting detrimental effects on environmental ecosystems. This has contributed to reduced soil fertility and an escalation in pest resistance to pesticides [3]. Consequently, there is mounting international emphasis on exploring agricultural production models that prioritize nutrient recycling and resource efficiency to ensure a sustainable supply of food products without inflicting severe ecological damage [4]. Integrated rice–fish farming is considered a sustainable agricultural production model that fosters symbiotic relationships between plants and animals, enhancing efficiency through nutrient recycling and reducing negative environmental impacts. This study aims to bridge the current knowledge gap regarding the optimal farming conditions of co-culture systems by exploring the effects of stocking density on the physiological state of common carp in a rice–fish co-culture system, contributing to the optimization of this sustainable agricultural practice.

Integrated rice–fish farming has gained widespread recognition as a beneficial approach for enhancing agricultural biodiversity and advancing agroecology, which not only supplies humans with both grain and meat but also diminishes the risk of environmental pollution [5]. In this system, rice paddies offer shade and water, creating a favorable habitat for fish, while weeds, plankton, and pests in the paddy field serve as food for the cultivated aquatic animals [6]. Furthermore, the natural predation of weeds and pests by farmed animals in this system can lead to a reduction in pesticide and herbicide use. Simultaneously, rice plants can absorb the nutrients from animal manure, thereby decreasing the necessity for synthetic fertilizers [7]. The synergy between different species and their complementary use of nutrients enhance the complexity of the rice–fish co-culture ecosystem. For example, the food chain within the system is extended and the stability of the system is enhanced, which means that the system is also more resistant to external environmental changes [8]. The rice–fish co-culture is also recognized as an effective way to maintain biodiversity and has great potential to reduce greenhouse gas emissions [9]. In recent years, owing to the good ecological and economic benefits of integrated rice–fish farming, it has expanded rapidly worldwide [10]. Specifically, in China, it has emerged as a primary mode of food production [11]. In 2022, the practice encompassed an area of approximately 2.86 million hectares, yielding around 21.5 million tons of rice and contributing to an aquatic production of 3.8722 million tons [10].

Driven by economic interests and increasing demand for food protein, the stocking density in aquaculture is generally maintained at a high level. This practice subjects farmed aquatic animals to several risks, including limited living space, competition for food, and social conflicts [12,13]. Thus, stocking density is a critical parameter in aquaculture [14]. For example, stocking density may affect the circadian rhythms and locomotor activity of African Catfish (*Clarias gariepinus*) [15]. Furthermore, stocking density can affect the mortality of infected fish; Chinook salmon (*Oncorhynchus tshawytscha*) infected with *Aeromonas salmonicida* exhibited increased mortality when reared at high density [16]. Numerous studies have demonstrated that an increase in stocking density negatively impacts the growth performance of aquatic animals, concurrently reducing their survival rate [17,18]. High stocking density exposes aquatic animals to crowded conditions and is, therefore, considered as a chronic stressor, resulting in an increased risk of infectious diseases and reduced immune function [19,20]. Previous studies have reported that high stocking densities can also cause oxidative stress and disorders of lipid and glucose metabolism [11,21]. In addition, high stocking densities significantly reduce the gut microbial diversity of aquatic animals, leading to the disruption of gut homeostasis and imbalance of microbial regulation [22].

The common carp (*Cyprinus carpio*), a member of the Cyprinidae family, primarily resides in freshwater settings such as ponds, lakes, and rivers [23]. It has a wide global distribution, particularly in Asia and several European countries [24]. In China, the common carp is a significant freshwater aquaculture species, with a cultivation yield reaching 2.843 million tons in 2022 [25]. The primary culture models for common carp include ponds and rice paddies in China. In ponds, high stocking density significantly reduced the weight gain of common carp [26]. Meanwhile, higher stocking density resulted in the elevation of glucose, free fatty acids, and lactate in the plasma of common carp [27]. However, in the rice–carp co-culture system, the effects of higher stocking densities on the carp’s antioxidant capabilities, nutrient composition, and metabolism function have yet to be examined. The current study, therefore, aimed to evaluate the impact of different stocking densities on the nutrient profile and metabolic functions in common carp muscle, employing multi-omics analysis.

## 2. Materials and Methods

### 2.1. Experimental System, Design, and Sampling

The experiment was conducted at the Jingjiang farming base of the Freshwater Fisheries Research Center (Jingjiang, China). The *C. carpio* utilized in the study were also provided by the farming base, having an average initial weight of 105.33 ± 2.52 g. The rice-fish farming system was constructed with an area of 20 m^2^ (5 m × 4 m). In this system, the variety of rice planted was Nangeng 5055, and the water depth was maintained at 10–20 cm.

In the rice–fish co-culture system, three stocking densities were established—low stocking density (LD, 52.9 g/m^2^), middle stocking density (MD, 105.8 g/m^2^), and high stocking density (HD, 158.7 g/m^2^)—with each density having three replicates. The fish were fed a commercial diet (Cargill Group Co., Ltd., Changzhou, China) containing 31% crude protein, 4% crude fat, 5.4% crude fiber, 18% crude ash, and 1% total phosphorus, once a day. The daily feeding amount was 1% of the fish’s weight. During the experiment, we conducted regular monitoring of the aquatic environment, as follows: the water temperature ranged from 18.5 to 30 °C, the dissolved oxygen level was between 3.2 and 5.8 mg/L, the pH value ranged from 6.8 to 7.6, total ammonia nitrogen was below 0.4 mg/L, and the nitrite nitrogen (NO_2_^−^-N) level was less than 0.05 mg/L. The paddy field was managed in accordance with traditional local agricultural practices (standard number SC/T 1135.1-2017) [28].

After a 60-day cultivation period, 15 fish were selected randomly from each group, following a 24 h fasting period. They were immediately anesthetized using MS-222 (50 mg/L, Sigma, St. Louis MO, USA) and, subsequently, white muscle tissue was obtained from their backs, specifically from the area between the head and the caudal peduncle, situated above the lateral line. Tissues from 5 fish were amalgamated into one sample for evaluating amino acid and fatty acid composition, as well as for conducting transcriptome and metabolome sequencing. It is important to highlight that to ensure sample stability, all specimens were initially stored in liquid nitrogen before being transferred to a −80 °C for prolonged preservation.

### 2.2. Assessment of Oxidative Stress Indicators

Oxidative stress markers, including superoxide dismutase (SOD), total antioxidant capacity (T-AOC), glutathione peroxidase (Gpx), glutathione (GSH), and malondialdehyde (MDA), were quantified using commercial kits. The T-AOC content was measured using the method reported by a previous study [29], while the MDA level was quantified following the method based on thiobarbituric acid (TBA) reactivity [30]. Gpx activity was assessed through a chromogenic reaction involving 5,5′-dithiobis (2-nitrobenzoic acid) (DTNB) and Cum-OOH as the substrate [31]. Similarly, the DTNB method was employed for determining the glutathione (GSH) level [32]. The SOD level was determined using the method reported by Peskin et al. [33], and total protein content was measured utilizing the method reported by Brown et al. [34].

### 2.3. Analyzing Amino Acids in Muscle

An appropriate quantity of muscle tissue was placed into a hydrolysis tube, to which 15 mL of 6.0 M hydrochloric acid solution was added. The tube was subsequently immersed in a cooling agent for 5 min and purged with nitrogen gas before securely tightening the cap. The sample was then hydrolyzed in a drying oven set at 110 °C for 22 h. Post-hydrolysis, the mixture was transferred into a 50 mL volumetric flask, and deionized water was added to reach the calibration mark. A 1.0 mL aliquot of filtrate was removed and placed into a 15 mL test tube, and then dried under vacuum at 40 °C to remove HCl. Subsequently, the residue was redissolved using sodium citrate buffer, following filtration via a membrane (0.22 μm); the supernatant was tested via an amino acid analyzer (Hitachi LA8080, Kyoto, Japan).

### 2.4. Determination of Fatty Acids in Muscle

An appropriate quantity of muscle tissue was subjected to hydrolysis using HCl (8.3 M, 70–80 °C, 40 min). The hydrolysate was then subjected to total lipid extraction using a mixture of diethyl ether and petroleum ether (1:1, *v*/*v*), in accordance with the protocol outlined in the Chinese national standard (GB5009.168-2016) [35]. For the conversion of the extracted lipids into methyl esters, methyl esterification was conducted using a 14% solution of boron trifluoride in methanol at 45 °C for 20 min. Analysis of the resulting fatty acid methyl esters (FAMEs) was conducted using an Agilent 6890 capillary gas chromatograph (GC) (Agilent Technology, Santa Clara, CA USA), which was equipped with a flame ionization detection (FID) system. Fatty acid composition was determined through comparison with standard fatty acid methyl esters (FAME) (Sigma, St. Louis, MO, USA).

### 2.5. Untargeted Metabolomic Profiling in Muscle

Approximately 100 mg of muscle tissue for each sample was ground in liquid nitrogen. The homogenate was then mixed with 80% methanol and diluted to a final concentration of 53%, followed by centrifugation at 15,000× *g* at 4 °C for 20 min to extract metabolites. The obtained supernatant was then utilized for LC-MS/MS measurement [36]. LC-MS/MS sequencing was conducted using a Vanquish UHPLC system (Thermo Fisher, Darmstadt, Germany) paired with an Orbitrap Q Exactive HF-X mass spectrometer (Thermo Fisher, Darmstadt, Germany). Subsequently, the raw date were analyzed using Compound Discoverer software (version 3.1), which conducted peak alignment, peak selection, and quantification of each metabolite. The metabolite characteristics were aggregated and normalized following standard protocols. Compounds demonstrating a relative standard deviation (RSD) exceeding 30% in quality control (QC) samples were excluded, enabling the achievement of metabolite identification and relative quantification. The identified metabolites were annotated by mapping them to the KEGG, HMDB, and LIPID MAPS database. The variances among samples were evaluated by principal component analysis (PCA). The metabolomics profile of the muscle between LD and HD groups was evaluated by orthogonal projection to latent structures-discriminant analysis (OPLS-DA). An independent sample t-test was performed to ascertain statistical significance between the LD and HD groups. Metabolites were classified as differential based on a variable importance in projection (VIP) score exceeding 1 and a *p*-value below 0.05.

### 2.6. Transcriptome Sequencing and Analysis

The total RNA from muscle samples of the LD and HD groups was isolated with the TRIzol kit (Invitrogen, Carlsbad, CA, USA). RNA purification involved enrichment from total RNA via poly-T oligo-attached magnetic beads, which subsequently facilitated the synthesis of cDNA. This cDNA served as the template for constructing libraries through PCR amplification. The libraries were sequenced on the Illumina Novaseq platform.

The raw data were processed by fastp software (version 0.20.0) to obtain clean reads [37]. The clean reads were mapped to the *Cyprinus carpio* reference genome (NCBI: GCF_000951615.1) via HISAT2 software (version 2.0.5) [38]. Each gene’s FPKM (Fragments Per Kilobase of transcript per Million mapped reads) value was derived by considering the length of the gene and the total number of reads aligned to it [39]. PCA was employed to investigate the correlation among different sample groups. The identification of DEGs between LD and HD groups was conducted via the DESeq2 R package (version 1.20.0) according to the threshold: change (|log_2_ FC|) of ≥1 and a *p*-value of <0.05 [40]. GO enrichment analysis and the KEGG pathway were realized by clusterProfiler software (version 3.8.1). A threshold of false discovery rate (FDR) ≤ 0.05 was set to determine significant enrichment of the GO term and KEGG pathway.

### 2.7. Quantitative Real-Time PCR Test

Total RNA from muscle samples of LD and HD groups was isolated by employing the RNAiso Plus kit (Takara, Beijing, China), chloroform, isopropanol, and ethanol purification. Following the assessment of quality and concentration, this RNA was utilized for cDNA synthesis through reverse transcription with a PrimeScript™ RT reagent kit (Takara), employing the two-step method. The synthesized cDNA was used as a template for conducting PCR with a TB Green Premix Ex Taq II kit (Takara). The acquired quantification cycle (Cq) values enabled the estimation of relative gene expression utilizing the 2^−∆∆Cq^ method, where β-actin was employed as the housekeeping gene. Details of the specific primers utilized in the current study are provided in Table 1.

### 2.8. Statistical Analysis

Preliminary data sorting was conducted using Excel software (version 2016), with further analysis conducted using SPSS software (version 27.0). Data were presented as means ± standard errors (SE). The Shapiro–Wilk test determined the normality of data distribution, while the Levene test checked for homogeneity of variances (Table S1). Differences in oxidative stress markers across different experimental groups were evaluated through one-way analysis of variance (ANOVA) and post hoc comparisons were analyzed using the least significant difference (LSD) test. The LD and HD groups’ amino acid and fatty acid profiles were compared using Student’s *t*-test. Additionally, the Pearson correlation test was applied to examine the relationship between RT-qPCR and RNA-seq data. Statistical significance was established at a *p*-value of less than 0.05.

## 3. Results

### 3.1. Alterations in Oxidative Stress Indicators

**Figure 1:**
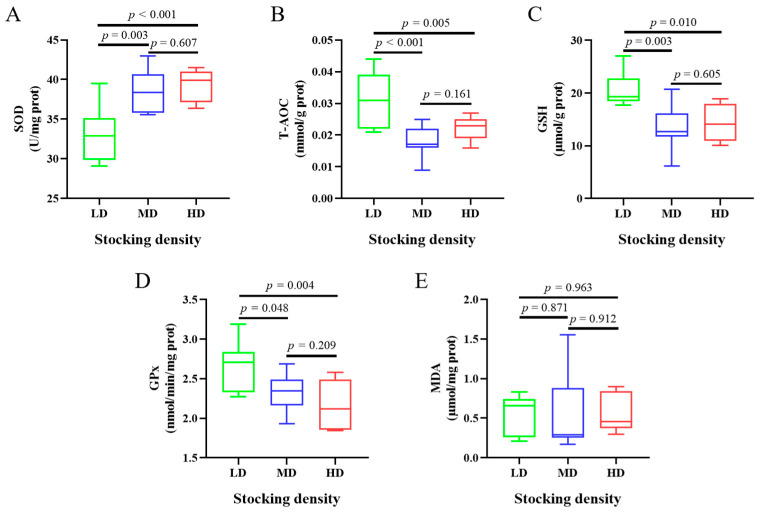
Oxidative stress indicators in muscles of *C. carpio* under different densities. LD, low stocking density; MD, middle stocking density; HD, high stocking density. (**A**) Superoxide dismutase (SOD); (**B**) Total antioxidation capacity (T-AOC); (**C**) Reduced glutathione (GSH); (**D**) Glutathione peroxidase (GPX); (**E**) Malondialdehyde (MDA).

The muscle antioxidant capability of common carp subjected to various stocking densities exhibited diverse changes (Figure 1). After 60 days of farming, there was a significant elevation in SOD activity under the MD and HD treatments when compared to the LD treatment (*p* < 0.05; Figure 1A). Conversely, the T-AOC, GSH, and GPx levels were significantly reduced in the MD and HD treatments relative to those in the LD group (*p* < 0.05; Figure 1B,C,D). Additionally, the MDA content across the different density groups did not show significant variation (*p* > 0.05; Figure 1E).

### 3.2. Alterations in Amino Acids Composition

The composition of amino acids in the muscle samples of *C. carpio* from both the LD and HD groups are presented in Table 2. Here, we identified seventeen amino acids, encompassing seven essential amino acids and eight non-essential amino acids. However, a significant difference was noted in the content of glycine, which decreased significantly with increasing stocking density (*p* < 0.05).

### 3.3. Alterations in Fatty Acids Composition

The muscle tissue of *C. carpio* was analyzed for fourteen fatty acids, which consisted of three saturated fatty acids (SFAs), four monounsaturated fatty acids (MUFAs), and seven polyunsaturated fatty acids (PUFAs). Notably, marked variations in fatty acid concentrations were detected between LD and HD groups (Table 3). In comparison to the LD group, levels of SFA (C14:0, C16:0, and C18:0), MUFA (C16:1, C18:1n9c, and C20:1), n-3 PUFA (C20:5n3), and n-6 PUFA (C18:2n6c and C20:3n6) exhibited a significant increase in the muscles of *C. carpio* in the HD group (*p* < 0.05). Moreover, the n-3/n-6 ratio showed a slight decline in the muscle of common carp stocked at HD relative to the LD treatment.

### 3.4. Metabolites Profile in Muscle

**Figure 2:**
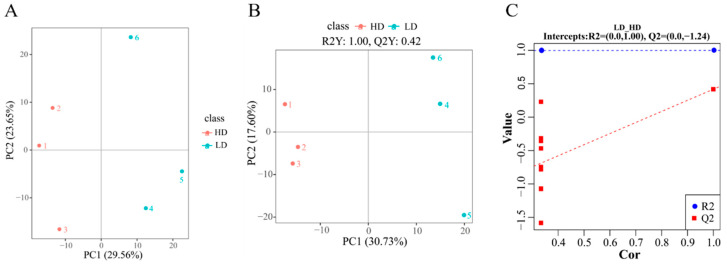
Analysis of metabolites in the muscles of *C. carpio* stocked at LD and HD. (**A**) PCA score plot of metabolites in the LD and HD groups; (**B**) PLS-DA score plots of metabolites in LD and HD groups; and (**C**) validation plot for PLS-DA model.

LC-MS analysis was utilized to explore the metabolic profiles of muscle samples from LD and HD groups. The unsupervised PCA yielded a distinct separation between these groups (Figure 2A), which was further supported by the clear classification observed through PLS-DA (Figure 2B). These results indicated significant differences in the metabolites of the groups subjected to varying density treatments. Additionally, results from cross-validation and permutation tests confirmed that the PLS-DA model was not subject to overfitting, thus reinforcing the model’s reliability (Figure 2C).

**Figure 3:**
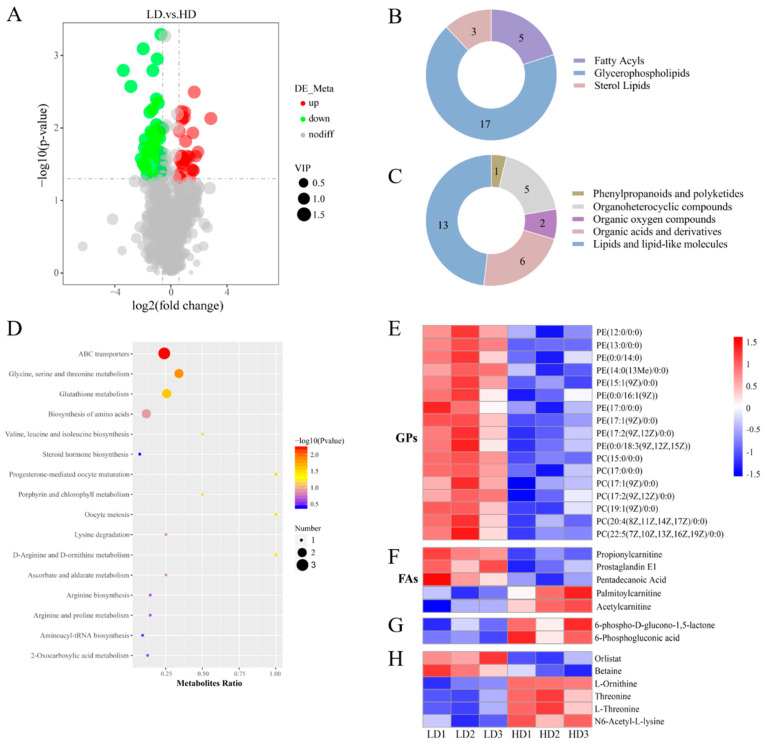
Metabolite profile in muscle of *C. carpio* stocked at LD and HD. (**A**) Volcano plot of the differential metabolites between LD and HD groups. (**B**) Numbers and classification of the differential metabolites via the LIPIDMAPS database annotation. (**C**) Numbers and classification of the differential metabolites via the HMDB database annotation. (**D**) KEGG pathways enriched by the differential metabolites. (**E**) Differential metabolites related to glycerophospholipids (GPs). (**F**) Differential metabolites related to fatty acyls (FAs). (**G**) Differential metabolites related to organic oxygen compounds. (**H**) Differential metabolites related to organic acids and derivatives.

A total of 914 metabolites were identified in the muscle tissue of *C. carpio*, among which 73 showed significant alterations. Compared with the LD group, the HD group demonstrated a notable reduction in 43 metabolites, while 30 metabolites showed significant elevation (Figure 3A). Based on the annotations from LIPIDMAPS, twenty-five distinct metabolites were identified, comprising five fatty acyls, three sterol lipids, and seventeen glycerophospholipids (Figure 3B). Annotations from HMDB revealed that the differential metabolites predominantly consisted of lipids and lipid molecules (13), organic acids and their derivatives (6), and organoheterocyclic compounds (5) (Figure 3C). KEGG enrichment analysis showed that the differential metabolites were primarily associated with ABC transporters (*p* = 0.006), glycine, serine, and threonine metabolism (*p* = 0.014), glutathione metabolism (*p* = 0.026), D-arginine and D-ornithine metabolism (*p* = 0.035), oocyte meiosis (*p* = 0.035), and progesterone-mediated oocyte maturation (*p* = 0.035; Figure 3D).

The metabolite annotation results showed significant changes in seventeen glycerophospholipids (GPs) and five fatty acyls (FAs) between the LD and HD groups. Among the GPs, HD treatment led to a significant decrease in ten PE and seven PC types. Specifically, the levels of PE(12:0/0:0), PE(13:0/0:0), PE(0:0/14:0), PE(14:0/0:0), PE(15:1/0:0), PE(0:0/16:1), PE(17:0/0:0), PE(17:1/0:0), PE(17:2/0:0), PE(0:0/18:3), PC(15:0/0:0), PC(17:1/0:0), PC(17:1/0:0), PC(17:2/0:0), PC(19:1/0:0), PC(20:4/0:0), and PC(22:5/0:0) were significantly lower in the HD group compared to the LD group (*p* < 0.05; Figure 3E). In FAs, the HD treatment significantly increased levels of palmitoylcarnitine and acetylcarnitine, but decreased levels of propionylcarnitine, prostaglandin E1, and pentadecanoic acid (*p* < 0.05; Figure 3F).

In addition, the levels of two organic oxygen compounds in the HD group, including 6-phospho-D-glucono-1,5-lactone and 6-phosphogluconic acid, were significantly increased compared to the LD group (*p* < 0.05; Figure 3G). As depicted in Figure 3H, the HD treatment caused changes in several organic acids and derivatives. Specifically, in comparison to the LD group, the levels of orlistat and betaine displayed an apparent decrease in the muscle of common carp with HD treatment, whereas the levels of L-ornithine, threonine, and N6-Acetyl-L-lysine showed a significant increase (*p* < 0.05).

### 3.5. Transcriptome Profile in Muscle

The sequencing data are presented in Table 4. After filtering the raw reads, we obtained over 40,071,582 clean reads. The Q_20_ and Q_30_ values ranged from 97.12% to 97.63% and from 92.41% to 93.44%, respectively, while the GC content varied between 48.66% and 49.44%. These results indicate that the sequencing quality of the muscle samples from the *C. carpio* is reliable.

**Figure 4:**
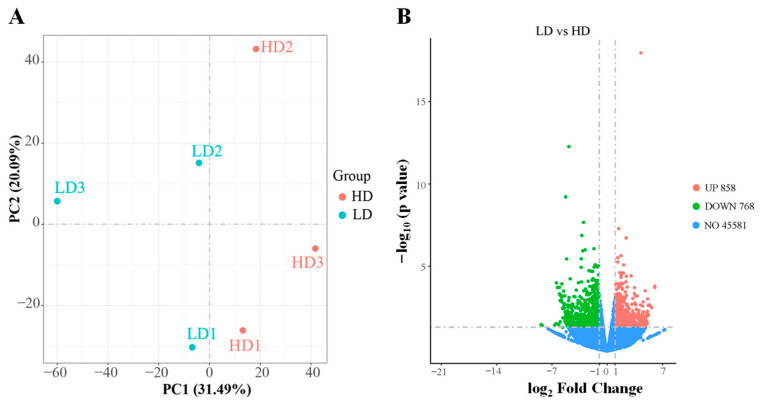
Differently expressed genes (DEGs) in the muscle of *C. carpio* stocked at LD and HD. (**A**) PCA plot of DEG between the LD and HD groups. (**B**) Volcano plot of DEGs between the LD and HD groups.

The results of PCA showed the formation of two distinct groups, demonstrating a clear distinction between the LD and HD treatments (Figure 4A). Furthermore, there were 1626 DEGs between the LD and HD treatments, with 858 downregulated genes and 768 upregulated genes in HD treatments (Figure 4B).

### 3.6. Enrichment Analysis of Gene Ontology (GO) and Kyoto Encyclopedia of Genes and Genomes (KEGG) 

**Figure 5:**
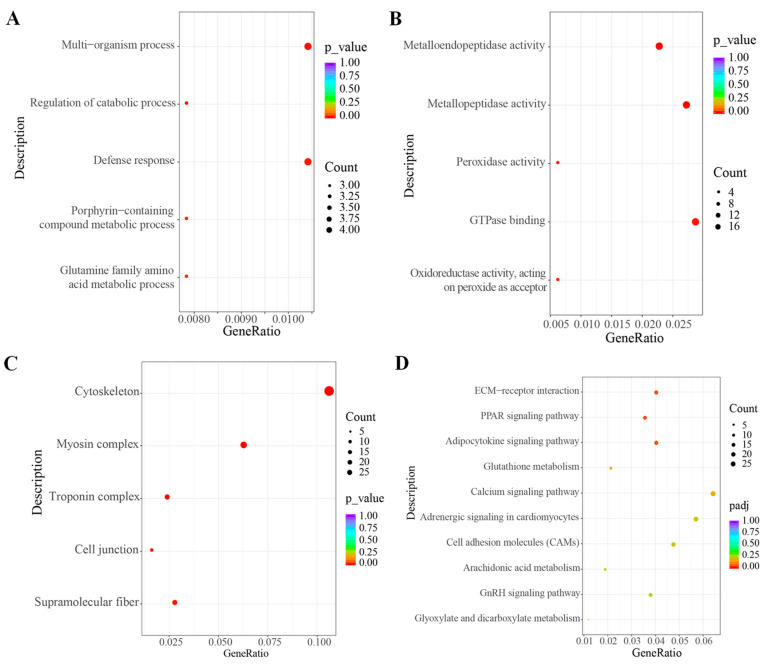
GO enrichment analysis and KEGG enrichment analysis on the differently expressed genes (DEGs) in the muscles of *C. carpio* between the LD and HD groups. (**A**) The top 5 altered GO terms (Level 2) in the biological process category following HD treatment compared with LD treatment. (**B**) The top 5 altered GO terms (Level 2) in the molecular function category following HD treatment compared with LD treatment. (**C**) The top 5 altered GO terms (Level 2) in the cellular component category following HD treatment compared with LD treatment. (**D**) The top 10 enriched KEGG pathways following HD treatment compared with LD treatment.

To enhance comprehension of how different stocking densities influence the biological functions of *C. carpio*, GO enrichment analysis was conducted on DEGs. In the biological process category, the DEGs were primarily involved in the multi-organism process, regulation of catabolic process, defense response, and glutamine family amino acid metabolic process (Figure 5A). In the molecular function category, the most enriched GO pathways for DEGs were metalloendopeptidase activity, metallopeptidase activity, and peroxidase activity (Figure 5B). In the cellular components category, the DEGs were mainly enriched in cytoskeleton, myosin complex, and troponin complex (Figure 5C).

According to KEGG enrichment analysis, the top 10 enriched pathways between LD and HD groups are listed in Figure 5D. The results indicate that DEGs in the enriched pathway were mainly associated with pathways related to lipid metabolism, including the PPAR signaling pathway, adipocytokine signaling pathway, and arachidonic acid metabolism. In addition, key cellular interaction pathways such as ECM-receptor interaction and cell adhesion molecules (CAMs) have been identified in the KEGG enrichment pathways.

### 3.7. Alterations of Key Signaling Pathways

**Figure 6:**
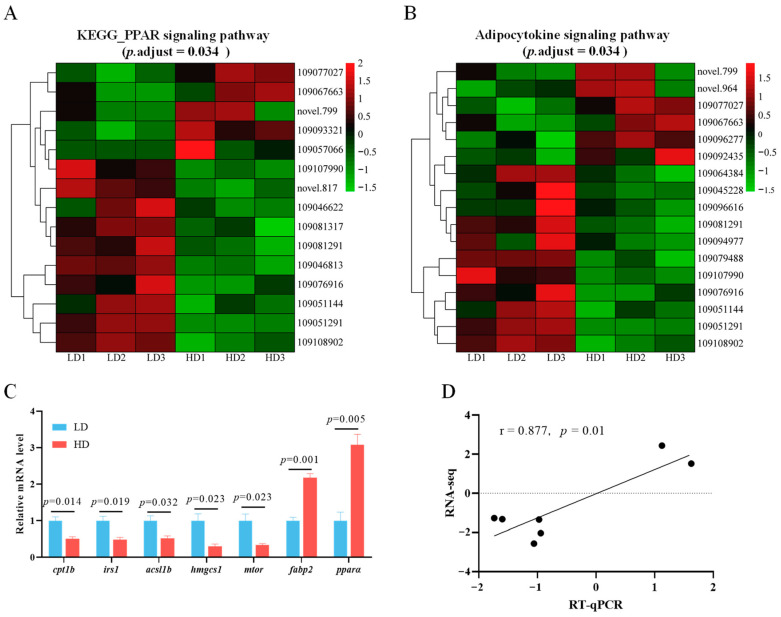
Expression of genes related to the PPAR signaling pathway (**A**) and the adipocytokine signaling pathway (**B**) in the muscles of *C. carpio* stocked at LD and HD. (**C**) Key gene expressions measured by RT-qPCR, with values expressed as the mean ± SE (n = 3). (**D**) Correlation between the RT-qPCR and RNA-seq data.

The PPAR signaling pathway in muscle tissue exhibited significant alterations in response to varying stocking densities (*p*.adj = 0.034; Figure 6A). In the HD group, compared to the LD group, five genes were upregulated and ten genes were downregulated. The upregulated genes included *pparα* (109077027) and *fabp2* (109093321), while the downregulated genes included *lpl* (109046622), *fabp1a* (109046813), *acsl1b* (109081291), and *hmgcs1* (109081317).

It is noteworthy that HD treatment also significantly impacted the adipocytokine signaling pathway (*p*.adjust = 0.034; Figure 6B). In the treatment with HD, six genes exhibited upregulation, while eleven genes showed downregulation in the adipocytokine signaling pathway. Among them, the expression of *irs1* (109045228), *mtor* (109094977), and *cpt1b* (109108902) was obviously downregulated in the HD group.

To validate the accuracy of the transcriptomic data, key genes from the PPAR and adipokine signaling pathways were randomly chosen for RT-qPCR analysis. This verification revealed a significant positive correlation between the RT-qPCR results and the RNA-seq data, thus substantiating the dependability of the transcriptomic findings (r = 0.877, *p* = 0.01; Figure 6C,D).

## 4. Discussion

High stocking densities are prevalent in aquaculture, enhancing space and resource utilization in production systems. However, increasing research indicates that excessive stocking densities may act as a stressor, inducing oxidative stress or damage in aquaculture species, which detrimentally impacts their health and physiological functions [11,20,41]. To mitigate the potentially detrimental effects of oxidative stress, fish have evolved defense mechanisms that encompass both enzymatic antioxidants such as SOD, GPx, and CAT, and non-enzymatic antioxidants, including GSH, vitamin C, and vitamin E. These defenses are crucial for neutralizing oxidative damage and maintaining physiological health [42]. Additionally, if antioxidant levels do not adequately counterbalance ROS production, it can result in DNA damage, protein oxidation, lipid peroxidation, and possibly apoptosis [43]. Previous research has demonstrated that high stocking density can elevate antioxidant marks including GSH, GPx, and T-AOC in *Cherax quadricarinatus*, thereby activating the oxidative defense system as a positive adaptive response to adverse conditions [44]. Similarly, a study of juvenile *Trachinotus ovatus* reared in off-shore sea cages showed that high stocking densities significantly increased serum levels of GPx and T-AOC [41]. Conversely, our study indicated that in the HD group, GPx and T-AOC activity, along with GSH content in the muscle of *C. carpio*, were significantly reduced after 60 days of rearing. Similar to our findings, high stocking density significantly reduced the antioxidant parameters of *Carassius gibelio*, including GSH, GPx, and T-AOC, compromising the antioxidant defense system [45]. Meanwhile, a previous study on juvenile turbot (*Scophthalmus maximu*) also supported our results [17]. The reduction in antioxidant parameters in farmed fish represents a severe oxidative response to stress from overcrowding. Once the tolerance limit is exceeded, this can lead to antioxidant depletion and impaired functioning of the antioxidant defense system [46,47]. 

High levels of free radicals can initiate chain reactions that oxidize polyunsaturated phospholipids, thereby impairing membrane function and causing lipid peroxidation [48,49]. The MDA, which is the final product of lipid peroxidation, frequently serves as a marker for oxidative stress levels [50]. Previous research has indicated that high stocking densities can act as a stressor, leading to increased MDA levels in various tissues of aquatic animals [11,44,51]. However, our study showed that stocking density did not have a clear impact on the formation of MDA in the muscles of *C. carpio*. We speculate that this may be due to the strong tolerance of muscles to lipid peroxidation caused by density stress.

The nutrient-rich composition of fish, characterized by high-quality protein and beneficial fats, is crucial for human health. The amino acid profile significantly influences the nutritional value of fish muscle. Early studies have indicated that stocking density can affect muscle protein and ash levels in fish [18], but investigations into how stocking density influences amino acid profiles in fish muscles are scarce. The results of the present study revealed that different densities did not cause significant changes in the amino acid composition in the muscle tissue of *C. carpio*. Our results were analogous to the data found in *Micropterus salmoides* [52]. However, the metabolome results revealed that HD treatment clearly impacted the levels of organic acids and their derivatives in the muscle. Specifically, there was a notable increase in the levels of four amino acids and their analogs, such as N6-acetyl-L-lysine, L-ornithine, threonine, and L-threonine, while levels of orlistat and betaine decreased significantly in HD group. N6-acetyl-L-lysine is a lysine that has been post-translationally modified [53]. It is well known that lysine, as an important essential amino acid, widely recognized for its crucial role in stimulating protein synthesis [54]. Threonine contributes to the breakdown of fat during metabolism, while a decrease in carcass fat is often related to the maintenance of amino acid balance [55]. Thus, the elevated levels of these amino acids and their analogs may potentially help carp cope with adverse environmental conditions.

Fatty acids are essential in activating stress response elements and preserving metabolic balance [56]. In fish, it has been observed that when the stocking density reaches a certain threshold, there is a notable decrease in fat content [18,41]. However, in this study, the levels of SFAs, such as myristic acid (C14:0), palmitic acid (C16:0), and stearic acid (C18:0), increased with rising stocking density. In line with our findings, the high-stocking-density treatment also led to a significant elevation in SFA content in the muscles of *Micropterus salmoides* [52]. We hypothesize that the significant increase in SFAs observed in the current study may be due to chronic stress resulting from high stocking densities. The process of lipolysis involves the breakdown of fats into glycerol and free fatty acids (FFAs). Numerous studies have confirmed that stressful conditions lead to metabolic alterations characterized by increased lipolysis [57,58]. Additionally, metabolomic analysis displayed that HD treatment markedly influenced the levels of various fatty acyls, particularly three carnitines. Specifically, the levels of palmitoylcarnitine and acetylcarnitine were markedly increased in the HD group, whereas propionylcarnitine levels were notably reduced. Previous studies have found that the roles of the above metabolites are involved in fatty acid metabolism, energy supply, and antioxidant and metabolic regulation, which are important for maintaining fish health and function [59,60,61]. The alterations in metabolite levels may reflect the adaptive response of *C. carpio* to crowding stress, although further research is necessary to elucidate the specific underlying mechanisms.

Numerous researchers have demonstrated the significant role of n-3 and n-6 PUFAs in maintaining membrane function homeostasis, impacting membrane fluidity, signaling processes, and gene regulation [62,63,64]. It should be noted that the findings of the current study revealed an obvious increased tendency in n-3 and n-6 PUFAs in the HD group compared to the LD group. Similar results were also observed in the muscle of grass carp (*Ctenopharyngodon idellus*) treated with high stocking density [65]. A high proportion of PUFAs in food offers significant health benefits, such as enhanced fetal development, improved brain health, and a reduced risk of coronary heart disease [66]. Fish in the HD group might be more beneficial due to their higher content of PUFAs. However, in the HD group, carp exhibited diminished growth performance [67], significantly reducing yields in the rice–fish co-culture system. Meanwhile, we speculate that under stress, a fish’s metabolism may undergo changes that could impact the synthesis and degradation of fatty acids, potentially resulting in increased levels of unsaturated fatty acids. Additionally, under oxidative stress, the release of more unsaturated fatty acids could enhance the antioxidant defense system. However, the specific mechanisms underlying these processes require further investigation.

Phosphatidylcholine (PC) and phosphatidylethanolamine (PE) are crucial constituents of cellular membranes, playing a pivotal role in diverse cellular functions and biological processes [68]. They are indispensable not only for upholding cell structure and function, but also for regulating lipid, lipoprotein, and energy metabolism [69,70]. Previous research has demonstrated that heat stress leads to significant reductions in the levels of serum PC (18:0/20:4), PC (15:0/23:4), PC (18:0/22:6), PC (18:2/18:2), and PE (18:1/18:1) in chickens [71]. Similar to the previous findings, high-stocking-density treatment resulted in decreased levels of ten types of PC and seven types of PE in muscle tissue. Furthermore, there have been reports indicating that the content of PC and PE affects the formation and growth of lipid droplets, in which reduced PE levels may prevent the aggregation of lipid droplets in muscle tissue [70,72]. Additionally, it has been observed that in muscle tissue, alterations is PC and PE are strongly linked to the formation of volatile flavor compounds [73]. These findings imply that HD treatment may decrease the likelihood of fat accumulation in muscle tissue, while also influencing its volatile flavor.

The PPAR signaling pathway is a key group of nuclear receptor pathways, with PPARα being a notable subtype within the PPAR family [74]. PPARα is widely recognized as a critical regulator of lipid metabolism, mediating processes such as fatty acid catabolism, lipogenesis, and ketogenesis through the regulation of various target genes [75]. Some studies have shown that activation of PPARα has a crucial role in preventing steatosis and in reducing inflammation and fibrosis [76]. High rearing density has been reported to suppress the expression of PPARα, resulting in disrupted lipid metabolism [11]. Similarly, the downregulation of *ppar*α in *Pelteobagrus fulvidraco* raised in high stocking densities points to abnormalities in lipid metabolism [77]. However, it is also interesting to note that contrasting results have been reported in *Acipenser schrenckii*, where high-stocking-density treatment upregulated PPARα to enhance lipid mobilization and utilization, thereby adapting to the unfavorable conditions [78]. In this study, high-stocking-density treatment resulted in the upregulation of *ppar*α and *fabp2*, but it also inhibited downstream genes such as *lpl*, *fabp1a*, *acsl1*, and *hmgcs1* in the PPAR signaling pathway. Therefore, significant alterations in PPAR signaling pathways induced by different stocking densities may offer insights into lipid metabolism disorders.

The adipokine signaling pathway is a critical intercellular communication system that involves the release and interaction of various adipokines. It plays diverse and essential biological roles, including regulating lipid metabolism, maintaining energy balance, and modulating immune responses [79]. Numerous studies have reported the detrimental effects of environmental stressors on the adipokine signaling pathway in aquatic animals. For instance, exposure to bisphenol A (BPA) in zebrafish offspring significantly enriched the adipocytokine signaling pathway, which resulted in disrupted lipid homeostasis [80]. Responding to salinity stress modulated key genes within the adipocytokine signaling pathway to maintain energy homeostasis [81]. Moreover, research has shown that high stocking density adversely affects the adipocytokine signaling pathways in *Micropterus salmoides*, leading to disorders in lipid metabolism [11]. In the current study, KEGG analysis exhibited significant alterations in the adipocytokine signaling pathway, with six genes being upregulated and eleven genes being downregulated. This indicates that high stocking density induces detrimental alterations in the adipocytokine signaling pathway in the muscle tissue, which further affects lipid metabolism.

## 5. Conclusions

In the rice–fish co-culture system, high stocking density, as a chronic stressor, resulted in the impairment of the antioxidant defense system in the muscles of *C. carpio*. With the increase in stocking density, there was a significant increase in fatty acids levels in the muscles of *C. carpio*, particularly in SFAs, n-3 PUFAs, and n-6 PUFAs. Metabolomic analysis revealed a reduction in phospholipid levels and an elevation in amino acid and its analog levels, as well as significant changes in various carnitine levels in the muscles of *C. carpio* after 60 days of high density farming. These observed alterations in metabolites may suggest an adaptive response of *C. carpio* to adverse conditions. Furthermore, the transcriptome results demonstrated that HD treatment led to significant alterations in the PPAR signaling pathway and adipokine signaling pathway, thereby impacting the lipid metabolism of *C. carpio*. The results from the current study offer meaningful insights for investigating the adaptation mechanism of carp to crowding stress in rice–fish co-culture systems.

## Figures and Tables

**Table 1:** The primer sequences for RT-qPCR in the present study.

Gene	Primer Sequence (5′-3′)
β-actin	F: ATCCGTAAAGACCTGTATGCCA
	R: GGGGAGCAATGATCTTGATCTTCA
carnitine palmitoyltransferase 1B (*cpt1b*)	F: GAAGAAACGTGCCATACGCTT
	R: AGGATGGCACTCAACACCG
insulin receptor substrate 1 (*irs1*)	F: GAGTGAGGAGTTCAGGCCAC
	R: AACGAATGGCTGTGTTTGCC
acyl-CoA synthetase long chain family member 1b (*acsl1b*)	F: CAGCAGTGTGGCATCGACAT
	R: GGGGCAGGTAAGAGATGTGTGT
3-hydroxy-3-methylglutaryl-CoA synthase 1 (*hmgcs1*)	F: AAGAAGGTTTCGCCCGATGT
	R: CAGGAAGTGATGAGTCCGGC
mechanistic target of rapamycin kinase (*mtor*)	F: TGGGTGTTTCTTTTCCCTCGT
	R: TGGGTTGTTTCTCCCAGGTC
fatty acid binding protein 2 (*fabp2*)	F: TCAGCACTTTCCGCACACT
	R: TTTCCGTTGTCCTTGCGTGT
peroxisome proliferator-activated receptor alpha (*pparα*)	F: GGGCGGATACCCCAATCTGA
	R: GCGTGCTTTGGCTTTGTTCA

**Table 2:** Hydrolyzed amino acid composition (g/100 g wet weight) in muscle of *C. carpio* stocked at low density (LD) and high density (HD).

Amino Acids (g/100 g)	Groups		
	LD	HD	*p*-Value
Threonine	0.67 ± 0.03	0.64 ± 0.02	*p* = 0.386
Valine	0.74 ± 0.03	0.70 ± 0.02	*p* = 0.268
Methionine	0.39 ± 0.02	0.39 ± 0.01	*p* = 0.936
Isoleucine	0.67 ± 0.03	0.63 ± 0.02	*p* = 0.293
Leucine	1.23 ± 0.06	1.17 ± 0.04	*p* = 0.378
Phenylalanine	0.68 ± 0.03	0.64 ± 0.02	*p* = 0.340
Lysine	1.52 ± 0.07	1.44 ± 0.05	*p* = 0.384
EAA	5.90 ± 0.26	5.60 ± 0.19	*p* = 0.372
Histidine	0.58 ± 0.03	0.53 ± 0.02	*p* = 0.235
Arginine	0.89 ± 0.04	0.84 ± 0.03	*p* = 0.297
Aspartic acid	1.49 ± 0.08	1.40 ± 0.05	*p* = 0.373
Serine	0.51 ± 0.02	0.48 ± 0.02	*p* = 0.189
Glutamic acid	1.92 ± 0.10	1.80 ± 0.08	*p* = 0.355
Glycine	0.82 ± 0.04	0.70 ± 0.02	*p* = 0.028
Alanine	0.96 ± 0.04	0.89 ± 0.03	*p* = 0.210
Cystine	0.15 ± 0.02	0.15 ± 0.01	*p* = 0.878
Tyrosine	0.47 ± 0.02	0.47 ± 0.02	*p* = 0.943
Proline	0.52 ± 0.02	0.46 ± 0.01	*p* = 0.064
NEAA	6.85 ± 0.32	6.36 ± 0.22	*p* = 0.247
TAA	14.22 ± 0.65	13.33 ± 0.46	*p* = 0.292

EAA, essential amino acid; NEAA, non-essential amino acid; TAA, total amino acid. All values are expressed as the mean ± SEM (*n* = 5).

**Table 3:** Hydrolyzed fatty acid composition (mg/100 g wet weight) in muscle of *C. carpio* stocked at low density (LD) and high density (HD).

Fatty Acids (mg/100 g)	Groups		
	LD	HD	*p*-Value
C14:0	4.3 ± 0.29	5.5 ± 0.28	*p* = 0.015
C16:0	101.9 ± 3.09	131.5 ± 5.89	*p* = 0.004
C18:0	37.0 ± 1.19	48.2 ± 1.78	*p* < 0.001
SFA	143.2 ± 4.13	185.2 ± 7.59	*p* = 0.001
C16:1	14.3 ± 0.95	19.9 ± 1.91	*p* = 0.032
C18:1n9c	111.0 ± 5.69	158.7 ± 12.09	*p* = 0.007
C20:1	6.6 ± 0.36	10.6 ± 1.17	*p* = 0.012
C22:1n9	4.0 ± 0.17	5.0 ± 0.66	*p* = 0.249
MUFA	135.1 ± 6.38	194.1 ± 14.43	*p* = 0.006
C20:2	4.2 ± 0.21	4.9 ± 0.19	*p* = 0.025
C18:3n3	7.4 ± 0.44	8.7 ± 0.51	*p* = 0.095
C20:5n3	10.0 ± 0.31	12.7 ± 0.67	*p* = 0.007
C22:6n3	61.5 ± 2.62	75.1 ± 5.52	*p* = 0.057
C18:2n6c	74.4 ± 4.82	100.9 ± 5.29	*p* = 0.006
C20:3n6	6.3 ± 0.43	8.4 ± 0.63	*p* = 0.024
C20:4n6	25.5 ± 1.42	25.9 ± 1.89	*p* = 0.850
PUFA	189.2 ± 7.25	236.6 ± 7.52	*p* = 0.002
n-3 PUFA	78.9 ± 2.86	96.4 ± 5.83	*p* = 0.027
n-6 PUFA	106.2 ± 5.57	135.2 ± 5.39	*p* = 0.006
n-3/n-6	0.75 ± 0.04	0.72 ± 0.06	*p* = 0.659

SFA, saturated fatty acid; MUFA, monounsaturated fatty acid; PUFA, polyunsaturated fatty acid. All values are expressed as the mean ± SEM (*n* = 5).

**Table 4:** Quality control of transcriptome sequencing.

Samples	Raw Reads	Clean Reads	Q_20_ (%)	Q_30_ (%)	GC (%)
LD-1	44196060	43603302	97.43	93.01	48.92
LD-2	40773658	40071582	97.63	93.44	49.19
LD-3	41394172	40804442	97.42	93.05	48.66
HD-1	43968464	43151978	97.45	93.13	49.29
HD-2	41525792	40895330	97.12	92.41	49.44
HD-3	46374674	45760408	97.36	92.91	49.23

Note: for Q_20_ and Q_30_, the base quality scores (Q scores) were no less than 20 and 30, respectively, in clean reads. GC, GC content in clean reads.

## Data Availability

The raw data of RNA-seq used in this study have been submitted to the open database NCBI Sequence Read Archive (PRJNA1110742). All data are contained within the main manuscript and Supplementary Material.

## Supplementary Materials

The following supporting information can be downloaded at: https://www.mdpi.com/article/10.3390/antiox13070849/s1, Table S1: Levene test and Shapiro–Wilk test for the data of oxidative stress markers before one-way analysis of variance (ANOVA).
