# Supplementary material for: Influences of Stocking Density on Antioxidant Status, Nutrients Composition, and Lipid Metabolism in the Muscles of Cyprinus carpio under Rice–Fish Co-Culture
Source: Antioxidants (Basel). 2024 Jul 15;13(7):849. doi: 10.3390/antiox13070849 (PMC11274104; doi:10.3390/antiox13070849)
Supplement: Supplementary file 1 [file antioxidants-13-00849-s001.zip › antioxidants-3036419-supplementary.pdf]

Table S1. Levene test and Shapiro-Wilk test for the data of oxidative stress markers before one-way analysis of variance (ANOVA)

Paramaters	Shapiro-Wilk test *p* value			Levene test *p* value
	LD	MD	HD	
SOD	0.6355	0.4214	0.5682	0.654
GSH	0.0699	0.8327	0.7266	0.854
Gpx	0.5404	0.9662	0.1374	0.656
MDA	0.2003	0.0667	0.2100	0.126
T-AOC	0.6979	0.5356	0.3147	0.062
